# Study on the Effect of Ultrasonic and Cold Plasma Non-Thermal Pretreatment Combined with Hot Air on the Drying Characteristics and Quality of Yams

**DOI:** 10.3390/foods14162831

**Published:** 2025-08-15

**Authors:** Xixuan Wang, Zhiqing Song, Changjiang Ding

**Affiliations:** 1 College of Science, Inner Mongolia University of Technology, Hohhot 010051, China; 202210908050@imut.edu.cn; 2 College of Electric Power, Inner Mongolia University of Technology, Hohhot 010080, China; ding9713@imut.edu.cn

**Keywords:** yam, hot-air drying, ultrasonic waves, drying characteristics, quality characteristics

## Abstract

In this study, the effects of non-thermal pretreatment such as corona discharge plasma (CDP-21 kV), dielectric barrier discharge plasma (DBDP-32 kV), and ultrasonic waves of different powers (US-180 W, 210 W, 240 W) on hot-air drying of ferruginous yam were compared. The regulatory effects of ultrasonic and cold plasma pretreatment on the drying characteristics and quality of yam were systematically evaluated by determining the drying kinetic parameters, physicochemical indexes, volatile components, and energy consumption. The results showed that ultrasonic pretreatment significantly improved the drying performance of yam compared with different cold plasma treatments, with the highest drying rate and effective moisture diffusion coefficient in the US-180 W group. In terms of quality, this treatment group exhibited better color retention, higher total phenol content (366 mg/100 g) and antioxidant activity, and optimal rehydration performance. Low-field nuclear magnetic resonance (NMR) analyses showed a more homogeneous water distribution, and gas chromatography–mass spectrometry (GC-MS) identified 55 volatile components. This study confirms that the US-180 W ultrasonic pretreatment technology can effectively improve the drying efficiency and product quality of yam and at the same time reduce the energy consumption. The results of this study provide a practical solution for the optimization of a process that can be replicated in the food drying industry.

## 1. Introduction

Yam (*Dioscorea opposita*), scientifically known as Dioscorea, refers to members of the genus Dioscorea in the family Dioscoreaceae. As a medicinal and food crop, it is rich in starch, polysaccharides, saponins, allantoin, and other functional components [1] and has a wide range of health benefits such as antioxidation, anti-inflammation, regulation of intestinal flora, hypoglycemic properties, and other health benefits, which have a wide potential for application in the food and pharmaceutical fields [2]. Due to its extremely high moisture content and susceptibility to microbial corrosion, yam is difficult to store for a long period of time [3]. Drying reduces the moisture to an optimal level, prolongs the shelf life, and inhibits the growth of microorganisms, which not only preserves the nutrients such as proteins, vitamins, and functional constituents such as antioxidant actives but also maintains the color of the product by inhibiting the browning reaction [4]. Therefore, it is necessary to choose the appropriate drying method according to the characteristics of the raw material.

The common methods of drying yams include freeze drying, microwave drying, far infrared radiation drying, and hot-air drying [5]. Freeze-drying retains heat-sensitive components and cell structure better, but is energy-intensive and costly [6]. Microwave drying heats moisture directly through electromagnetic radiation, significantly shortening the drying time, but there are limitations of uneven heating and the high cost of equipment [7]. Infrared drying has the advantages of fast heating and low energy consumption, but the parameter optimization requirements are high, which can easily lead to local overheating [8]. Hot-air drying, as a widely used drying method in the food industry, occupies more than 85% of the drying forms due to its flexible operation, low cost, simple equipment, and other advantages. However, there are significant drawbacks, as prolonged exposure to high temperatures can also lead to the loss of heat-sensitive substances such as vitamin C, polyphenolic compounds, and antioxidant active ingredients, which reduces the nutritional value and organoleptic quality of the final product [9,10]. Therefore, pretreatment before hot-air drying is essential to improve the drying efficiency and quality of yam.

The application of pretreatment technology in fruit and vegetable drying research has been expanding, and the common pretreatment methods nowadays include blanching, freeze–thaw, ultrasonic (US), dielectric barrier discharge plasma (DBDP), and corona discharge plasma (CDP) technologies. Blanching is performed by heat treatment to inactivate enzymes, which is effective in preventing browning but may lead to the degradation of heat-sensitive components [11]. Freeze–thaw uses ice crystals to disrupt the cellular structure, which improves drying efficiency, but excessive freezing and thawing may damage the texture [12]. In contrast, ultrasonic pretreatment, usually at lower temperatures, disrupts the cellular structure of food products by creating a cavitation effect that accelerates water migration and evaporation, while helping to retain vitamins and other nutrients in the food products. In a study by Wiktor et al., it was found that ultrasonic pretreatment significantly reduced drying time and improved retention of biologically active constituents (e.g., carotenoids) in carrots and apples [13]. There is definitely a role played by the pulsed electric field present in DC pulsed dielectric blocking discharge plasma, and it has been shown that a pulsed electric field works as a drying pretreatment by inducing the formation of micropores in plant cell membranes to enhance intra- and extracellular water transfer, thus improving the drying efficiency [14]. According to Du et al., DBDP pretreatment was found to significantly reduce the drying time of goji berries by 22.7% and improve the nutrient retention of the dried product—for example, by increasing the polysaccharide content [15]. Discharge plasma technology ionizes gas molecules by means of a high-voltage electric field, generating energetic particles, which in turn form a plasma that physically or chemically modifies the target [16]. Obajemihi et al. demonstrated that cold plasma pretreatment had a significant optimization effect on infrared-accelerated pulse-vacuum drying of tomato [17].

In this study, we systematically compared the effects of ultrasonic and cold plasma (CDP and DBDP) non-thermal pretreatment combined with hot-air drying on the drying characteristics and quality of yam, aiming at comparing the effects of US, DBDP, and CDP pretreatment combined with the hot-air drying of yam, and analyzed the changes in drying characteristics, nutritional quality, and volatile components of the yam, in order to provide new technical references for optimizing the drying pretreatment of yam and other foodstuffs.

## 2. Materials and Methods

### 2.1. Experimental Materials

The experimental material, yam, was purchased from a supermarket near Inner Mongolia University of Technology and stored in a refrigerator at −4 °C. Fresh Tiegun yams were peeled and cut into cylindrical slices with a thickness of 3 mm and a diameter of 12 mm for the experiment.

### 2.2. Experimental Content and Methods

#### 2.2.1. Pretreatment Experiments

**Figure 1:**
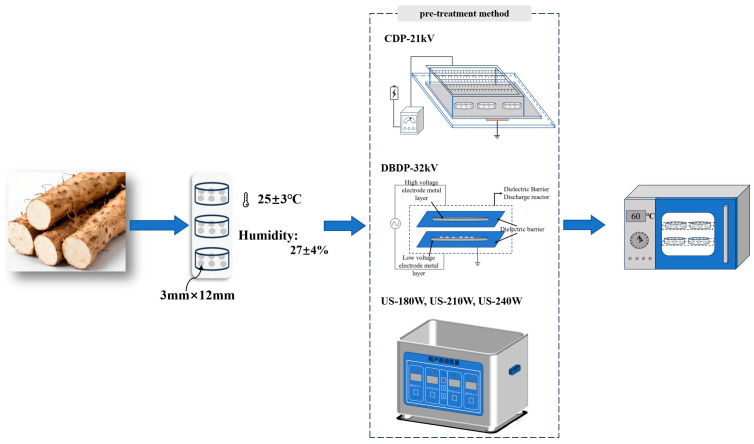
Schematic flow chart for the study of the effect of ultrasonic and cold plasma non-thermal pretreatment combined with hot air on the drying characteristics and quality characteristics of yams.

The experimental flow is shown in Figure 1. Three different pretreatments were applied to the yam slices before hot-air drying. The prepared yam slices were evenly placed in petri dishes, with each group weighing 12 ± 0.5 g. The Corona Discharge Plasma (CDP) device consists of a needle array-plate electrode system, a 4 mm thick dielectric plate covered by a grounded electrode, a high-voltage power frequency AC power supply, and a control system (YD (JZ)-1.5/50, Wuhan Boyu Electric Power Equipment Co., Ltd., Wuhan, China). The experiment parameters were set as follows: air gap distance of 4 cm and voltage of 21 kV. The petri dish containing the samples was placed on the lower electrode plate of the CDP device for pretreatment, the Dielectric Barrier Discharge Plasma (DBDP) device adopts a plate–plate DBD device with a bipolar high-voltage DC pulse power supply (P60D-V-ND, Dalian, China) adjustable from 0 to 40 kV, the upper and lower electrodes are circular with a diameter of 20 cm and a gap of 1 cm, the voltage used in the experiment was 32 kV, and the petri dish with samples was placed on the lower dielectric plate of the DBDP device for pretreatment. For ultrasonic (US)-assisted drying pretreatment, samples were placed in a beaker filled with deionized water and then treated in an ultrasonic processor (KQ-300DE, Kunshan, China). The temperature was set at 30 °C, and the power was 180, 210, and 240 W, respectively. The pretreatment was conducted under ambient conditions of 25 ± 3 °C and relative humidity of 28 ± 5%, with a pretreatment time of 30 min for all groups.

#### 2.2.2. Oven Drying

After 30 min of pretreatment, the yam slices were placed in an oven (DGX-9053, Shanghai Fangyuan Experimental Instrument Co., Ltd., Shanghai, China) with a continuously adjustable temperature range of 10 to 250 °C. The oven temperature was set to 60 °C, and the hot air velocity was 2 m/s. The samples were taken out every 30 min and weighed using an electronic balance (BS124S, Shanghai Guanglu Electronic Technology Co., Ltd., Shanghai, China) until the moisture content remained constant.

### 2.3. Drying Characteristics

#### 2.3.1. Moisture Content

The moisture content of yams during drying was determined by referring to Zhang et al. [18]. The dry basis moisture content and moisture ratio of yams are expressed as follows:

(1) $$M_{i}=\frac{m_{i}-m_{g}}{m_{g}}$$

(2) $$MR=\frac{M_{i}-M_{e}}{M_{0}-M_{e}}$$

where $m_{i}$ (g) is the real-time mass of yam during drying, $m_{g}$ (g) is the mass of dried yam, $M_{e}$ (g) is the equilibrium moisture content, $M_{0}$ (g) is the initial moisture content in yam, and 5 ± 0.5 g and $M_{i}$ (g) is the dry basis moisture content at a specific time. Since the equilibrium moisture content $M_{e}$ is much smaller than $M_{0}$ and $M_{i}$, $M_{e}$ can be neglected, and Equation (2) is simplified to:

(3) $$MR=\frac{M_{i}}{M_{0}}$$

#### 2.3.2. Average Drying Rate and Average Drying Time

The average drying rate (VR) represents the amount of water evaporated per unit mass of yam per unit time, which is expressed as:

(4) $$VR=\frac{m_{0}-m_{t}}{m_{0}T\;}$$

where $m_{0}$ (g) is the mass of yam before drying, $m_{t}$ (g) is the mass of yam after drying, and T is the drying time.

#### 2.3.3. Rehydration Rate

Dried yams were placed in a beaker containing 100 mL of deionized water and incubated in a 37 °C constant temperature water bath until the weight remained constant. After removal, the surface moisture of the yams was blotted with absorbent paper, and the weight was measured using an electronic scale (BS124S, Shanghai Guanglu Electronic Technology Co., Ltd., Shanghai, China). The calculation formula is as follows:

(5) $$RR=\frac{m_{b}}{m_{a}}$$

where $m_{b}$ (g) is the mass after soaking in the water bath, and $m_{a}$ (g) is the mass before rehydration.

#### 2.3.4. Effective Moisture Diffusivity

The effective moisture diffusivity of yams was determined based on Fick’s second law and nonlinear regression of experimental data, referring to the method by Corzo et al. [19]. The calculation method is as follows:

(6) $$\frac{dM}{dt}=D_{eff}\frac{d^{2}M}{dr^{2}}$$

For long-term drying with MR < 0, Equation (5) can be expressed as:

(7) $$lnMR=\frac{8}{\pi^{2}}-\frac{\pi^{2}D_{eff}t}{L^{2}}$$

where $D_{eff}$ (m^2^/s) is the effective moisture diffusivity, L (m) is half of the yam slice thickness, and t (s) is the drying time.

### 2.4. Color

An automatic colorimeter (3nh-NR60CP, Shenzhen, China) was used to directly measure the color changes before and after drying, including lightness ($L^{*}$), redness ($a^{*}$), and yellowness ($b^{*}$). Before measurement, the instrument was calibrated to ensure that the reference values met international color standards. Before and after drying, 5 evenly distributed points on the surface were selected for multi-point scanning to eliminate local color differences, and the final data were output as the average of five measurements. The total color difference (∆E) was calculated using the color parameters $L^{*}$, $a^{*}$, $b^{*}$ [20] with the formula:

(8) $$∆E=\sqrt{{\left(L_{i}^{*}-L_{0}^{*}\right)}^{2}+{\left(a_{i}^{*}-a_{0}^{*}\right)}^{2}+{\left(b_{i}^{*}-b_{o}^{*}\right)}^{2}}$$

where ∆E represents the color difference before and after drying; $L_{0}^{*}$, $a_{0}^{*}$, and $b_{o}^{*}$ are the colorimetric values of fresh yams; and $L_{i}^{*}$, $a_{i}^{*}$, and $b_{i}^{*}$ are the colorimetric values of dried yams. The whiteness of dried yams was measured to effectively evaluate the browning degree and quality stability [21], calculated as:

(9) $$Whiteness=100-\sqrt{{\left(100-L^{*}\right)}^{2}+{\left(a^{*}\right)}^{2}+{\left(b^{*}\right)}^{2}}$$

where $L^{*}$, $a^{*}$, and $b^{*}$ are the lightness, redness, and yellowness of dried yams.

### 2.5. Energy Consumption Analysis

Accurate measurement of energy consumption for drying materials is a core decision-making basis for optimizing production costs, improving energy efficiency, and achieving industrial sustainability. It was mainly measured by the power of cold plasma (CDP, DBDP), ultrasound, and hot-air drying. The calculation formula is as follows:

(10) W=P×t

where P is the power consumption of different pretreatment methods, and t is the time used for different pretreatment processing. Specific Energy Consumption (SEC) refers to the amount of energy required to remove a unit mass of water and is calculated as follows:

(11) $$SEC=\frac{W}{m_{0}-m_{g}}$$

where *SEC* (kJ/kg water) represents the specific energy consumption of drying, $m_{0}$ (g) is the mass of yam before drying, and $m_{g}$ (unit: g) is the mass of yam when drying is completed.

### 2.6. Reducing Sugar Content

The reducing sugar content was determined by the 3,5-dinitrosalicylic acid (DNS) colorimetric method. A glucose standard solution (1 mg/mL) was mixed with DNS reagent, heated in a boiling water bath for 5 min, and diluted to 25 mL, and the absorbance was measured at 540 nm to draw a standard curve. For sample determination, 3 g of sample was mixed with water to form a paste, infiltrated at 50 °C for 20 min, filtered, and 2 mL of the filtrate was taken, mixed with 1.5 mL of DNS reagent, and treated identically for color development and measurement. The content was calculated using the standard curve [22].

### 2.7. Total Phenol Content and Antioxidant Activity

The total polyphenol content of dried yams was determined by the Folin–Ciocalteu colorimetric method. The dried samples were crushed, and an appropriate amount of sample was mixed with a 70% acetone aqueous solution, subjected to ultrasonic treatment for 1 h, and centrifuged to collect the supernatant. The supernatant was mixed with diluted Folin–Ciocalteu reagent and 75 g/L sodium carbonate solution, reacted in the dark at room temperature for a certain period, and the absorbance was measured at 760 nm. The results were expressed as gallic acid equivalents (mg GAE/100 g dry weight or fresh weight) [23,24].

The antioxidant activity of dried yams was evaluated by determining their DPPH free radical scavenging capacity. The sample to be tested was mixed with a DPPH ethanol solution of known concentration, reacted in the dark at 25 °C for 30 min, and the absorbance at 517 nm was measured using an ultraviolet spectrophotometer (UV-1800, Thermo Fisher Scientific, Waltham, MA, USA). The remaining DPPH concentration was calculated according to the standard curve to evaluate the free radical scavenging capacity of the sample [25].

### 2.8. Low-Field NMR and Imaging

Low-Field Nuclear Magnetic Resonance (LF-NMR) analysis combined T2 relaxation spectrum and proton imaging technology. A nuclear magnetic resonance analyzer with a magnetic field strength of 0.5 T and a coil diameter of 60 mm was used to determine the transverse relaxation time (T2) of yams via the CPMG pulse sequence. The parameters included main frequency (SF), echo time TE = 0.15 ms, repetition time TW = 3000 ms, number of echoes NECH = 8000, and number of scans NS = 4. Moisture states were classified into bound water (T21: 0.1–10 ms), immobile water (T22: 10–100 ms), and free water (T23: 100–1000 ms), with their content proportions characterized by peak areas (A21, A22, A23). Meanwhile, proton density-weighted images were obtained based on the multi-slice spin echo (MSE) sequence, and moisture distribution was visually presented via pseudo-color mapping (red areas indicate high moisture content). These two methods synergistically quantified the dynamic characteristics of moisture migration and microstructural changes during drying, providing comprehensive technical support for real-time monitoring of moisture states in materials [26].

### 2.9. Determination of Volatile Components (GC-MS)

Volatile organic compounds (VOCs) in dried yams were determined by headspace solid-phase microextraction (SPME) combined with gas chromatography–mass spectrometry (HS-SPME-GC-MS) [27]. An amount of 2 g of dried yams was ground and placed in a headspace vial, equilibrated at 50 °C for 15 min, and then adsorbed using a DVB/CAR/PDMS fiber for 30 min. After desorption at 250 °C for 3 min, GC-MS analysis was performed. A DB-5MS column (30 m × 0.25 mm × 0.25 μm) was used, with helium as the carrier gas at a flow rate of 1.0 mL/min. The temperature program was as follows: initial temperature of 60 °C (held for 5 min) and increased to 280 °C (held for 5 min). The mass spectrometry conditions were ion source temperature of 230 °C, interface temperature of 280 °C, and scanning range of 45–450 m/z [28]. The relative content of each component was calculated by the peak area normalization method, and heptanone (8 ppm, 20 μL) was used as the internal standard for quantitative calibration.

### 2.10. Statistical Analysis

All experiments were repeated three times, and the results were expressed as mean ± standard deviation. One-way analysis of variance (ANOVA) was used to test the significance of differences between groups (*p* < 0.05). After data normalization, Origin 2021 software was used to generate heatmaps to analyze the correlation between different drying methods and various indicators, and the Pearson correlation coefficient matrix was used to explore the internal relationships between indicators. In addition, orthogonal partial least squares discriminant analysis (OPLS-DA) was used for multivariate statistical analysis of volatile components, with model fitting indices (R^2^ and Q^2^) both exceeding 0.5. These statistical methods collectively revealed the significant effects of drying technologies on sample characteristics and quality, providing solid mathematical support for the research conclusions [13,29].

## 3. Results and Discussion

### 3.1. Analysis of Drying Characteristics

**Figure 2:**
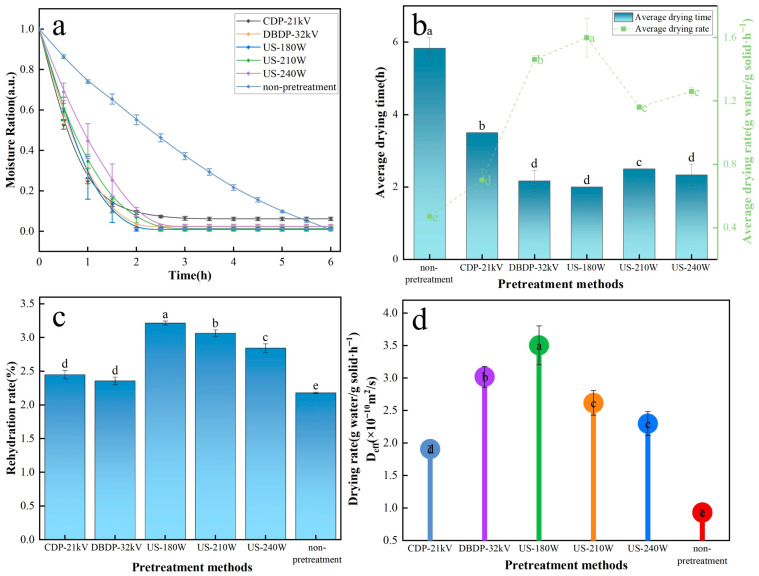
Curves of drying characteristics of yam with different non-thermal pretreatments combined with hot-air drying: (**a**) change in moisture content with time; (**b**) average drying time and average drying rate; (**c**) rehydration rate; (**d**) effective moisture diffusion coefficient. Different letters indicate significant differences between sample means.

Figure 2 shows the drying characteristics of yam under different treatment methods. Figure 2a,b show the effects of different treatment methods on the moisture content, average drying rate, and average drying time of yam after drying. Compared with no pretreatment, the average drying rates of CDP-21 kV, DBDP-32 kV, US-180 W, US-210 W, and US-240 W increased by 48.9, 67.8, 70.6, 59.5, and 62.6%, respectively. It was shown that both non-thermal pretreatments were effective in accelerating water evaporation and shortening the time for drying to reach constant weight. This accelerating effect stems from different mechanisms of action, with CDP promoting surface water evaporation through the generation of ionic wind by a high-voltage electric field, DBDP enhancing water penetration through the formation of micropores by surface etching, and the cavitation effect of ultrasound forming microchannels within the tissue, significantly reducing the resistance to water migration [14,30]. This is clearly reflected in the effective water diffusion coefficient results in Figure 2d, where the diffusion coefficient of the ultrasound group was higher than that of the plasma group as a whole, with the US-180 W group being the highest, further confirming its advantage in promoting water migration. The difference in rehydration performance is clearly presented in Figure 2c, where the rehydration rate of the ultrasound pretreated group was significantly higher than that of the plasma group (*p* < 0.05), with the US-180 W group showing the best performance, which was attributed to the micro-rupture of the cell wall due to ultrasonic cavitation, resulting in the formation of a porous structure that enhances the hydration capacity, which was verified from the study of mushrooms by Jambrak et al. [31]. Comprehensive data from each graph in Figure 2 shows that the US-180 W group has the best performance in terms of average drying rate, effective water diffusion coefficient, and rehydration rate, indicating that the ultrasonic pretreatment with moderate power can synergistically improve the drying efficiency and product water-holding performance through physical structure modification.

### 3.2. Analysis Color

**Figure 3:**
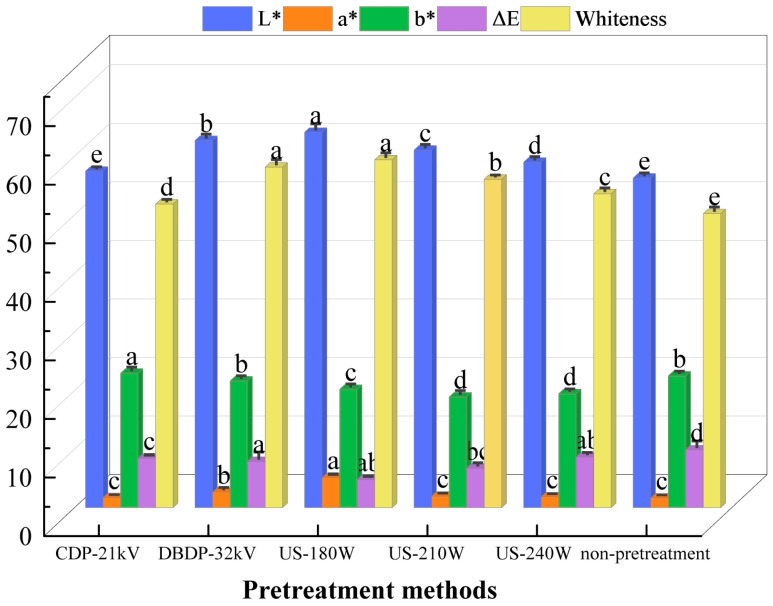
Color changes of yam after different non-thermal pretreatments combined with hot-air drying. L*: lightness value; a*: redness value; b*: yellowness value; *ΔE*: color difference; *Whiteness*: whiteness value. (Different letters indicate significant differences between sample means).

Color is a key index to evaluate the quality of dried materials. It is not only directly related to the appearance and processing performance of materials but also closely related to consumer acceptance. During the drying process, the color change of materials can intuitively reflect the chemical reactions occurring inside them (such as pigment degradation, Maillard reaction, etc.), so it is often used to evaluate the impact of the drying process on quality and provide an important basis for the optimization of drying technology [32]. Figure 3 shows the effect of different pretreatment methods on the color of dried yams. The results show that there are significant differences between groups (*p* < 0.05). Among them, the yam in the ultrasonic pretreatment group shows relatively high levels in lightness value (L*) and redness value (a*), and small color differences (*ΔE*). Generally speaking, the increase in lightness value (L*) and redness value (a*) of yam is related to non-enzymatic browning reactions such as the Maillard reaction, oxidation of phenolic substances, and degradation of ascorbic acid: under high temperature conditions, reducing sugars react with amino acids in the Maillard reaction to generate brown substances such as melanoidins, which leads to the increase in redness value; at the same time, the oxidation reaction between iron ions and polyphenols will further enhance redness. The high temperature (60 °C) in the hot-air drying process is the basic factor leading to the color change. On the one hand, the high temperature accelerates the Maillard reaction (the reaction between reducing sugars and amino acids to produce brown substances), leading to an increase in the redness value (a*); on the other hand, the high temperature directly destroys the heat-sensitive pigments, such as carotenoids, leading to a decrease in the yellowness value (b*) [33]. In addition, the volatilization of aldehydes and the generation of lipid oxidation products will also affect color changes.

In contrast, non-thermal treatment methods such as cold plasma give yams a higher lightness value (L*) and whiteness value and a relatively lower redness value (a*) due to reducing thermal reactions [34]. Unlike the negative effect of temperature, ultrasonic pretreatment (especially in the US-180 W group) can shorten the exposure time of the material in a high-temperature environment by accelerating the migration of water to the surface and rapid evaporation through the microchannels formed by the cavitation effect. At the same time, the mechanical vibration of ultrasound inhibits the activity of browning enzymes (e.g., polyphenol oxidase), which directly reduces the browning caused by oxidation of phenolics and ultimately reduces the total color difference [35]. For ultrasonic pretreatment with different powers, with the increase in power, some materials will have slight enzymatic browning, resulting in a decrease in the lightness value and redness value of the yam, while the yellowness value shows an increasing trend due to pigment changes [36]. Therefore, ultrasound does not change the temperature directly but reduces the effect of high temperature on the color by its own action (accelerated water migration to reduce exposure to high temperature; the inhibition of browning enzyme activity), which is the key to better color retention in the US-180 W group.

### 3.3. Drying Energy Consumption

**Figure 4:**
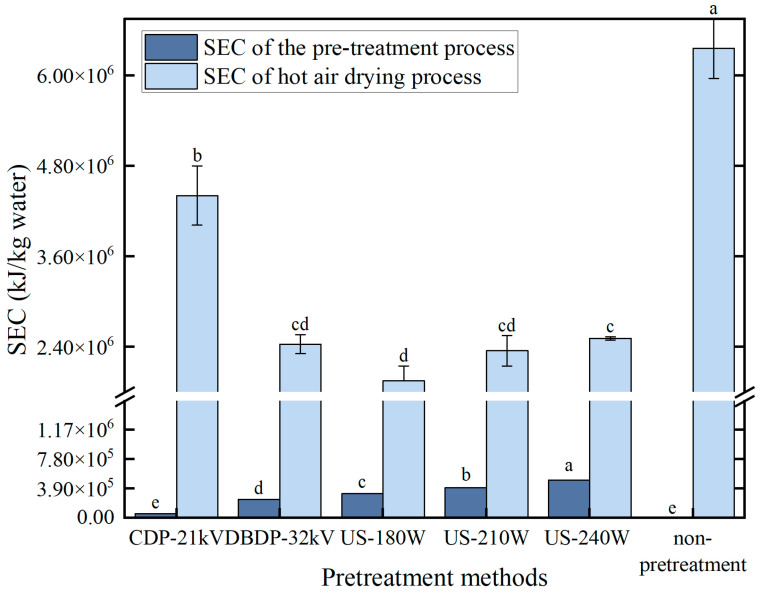
Energy consumption of different non-thermal pretreatments. (Different letters indicate significant differences between sample means).

The analysis of energy consumption shown in Figure 4 indicates that although the pretreatment energy consumption of the US-180 W group is higher than that of the cold plasma group, the energy consumption of hot-air drying is significantly lower, and the final total energy consumption is lower. The core reason for this is the difference in the effect of different pretreatments on drying efficiency. US-180 W pretreatment formed microchannels in the yam tissue through the ultrasonic cavitation effect, which significantly reduced the resistance to water migration and accelerated the diffusion of water to the outside, so that the hot-air drying time was greatly reduced. The energy consumption of hot-air drying was directly related to the time, so the energy consumption of the hot-air stage was significantly reduced in the US-180 W group.

On the contrary, the cold plasma treatment, although the pretreatment stage consumes less energy due to the power characteristics of the equipment, only forms micropores through surface etching, which promotes the migration of deep water more weakly than the microchannel effect of ultrasound, leading to the prolongation of the hot-air drying time. Although the energy consumption of the pretreatment is relatively low, extending the hot-air drying time leads to a significant increase in energy consumption during this stage. This result is consistent with the conclusions of Du et al., who found that the impact of pretreatment on subsequent drying time is a key factor determining the energy consumption of the drying process [10], thereby confirming the advantages of ultrasonic pretreatment in terms of energy efficiency.

### 3.4. Analysis of Reducing Sugar Content

**Figure 5:**
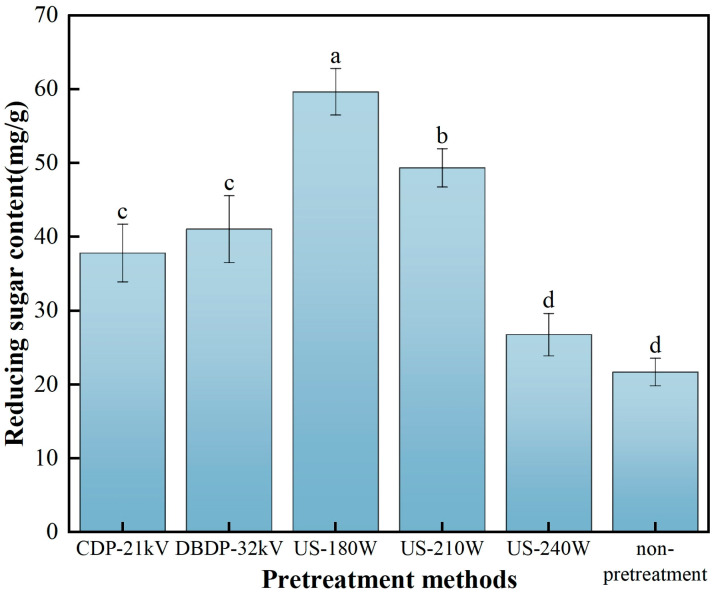
Reducing sugar content of yam after different non-thermal pretreatments combined with hot-air drying. (Different letters indicate significant differences between sample means).

The determination of reducing sugar content in dried materials is mainly used to evaluate the impact of the drying process on sugar retention, so as to ensure the quality stability of products in terms of nutrition, flavor, and color. In addition, by monitoring the change in sugar content during storage, the stability of products can be judged, providing reliable data support for production practice and scientific research [37]. Figure 5 presents the effect of different non-thermal pretreatment methods on the reducing sugar content in hot-air-dried yam. The research results show that compared with the CDP-21 kV treatment group, the reducing sugar contents in the US-180 W and US-210 W treatment groups increase by 36.58 and 23.34% respectively; compared with the DBDP-32 kV treatment group, the reducing sugar contents in these two groups increase by 31.17 and 17.42%, respectively. The above data indicate that ultrasonic pretreatment can effectively promote the retention of reducing sugar in yam, which is also verified by Bai et al.’s research on yam [22]. The retention of reducing sugars is of great significance to the quality of the product, not only directly imparting sweetness to enhance organoleptic acceptability and acting as a precursor in the production of flavoring substances but also influencing protein function and antioxidant properties through the Meladic reaction, which is essential for maintaining nutritional activity in yam processing [38].

### 3.5. Analysis of Total Phenol Content and Antioxidant Activity

**Figure 6:**
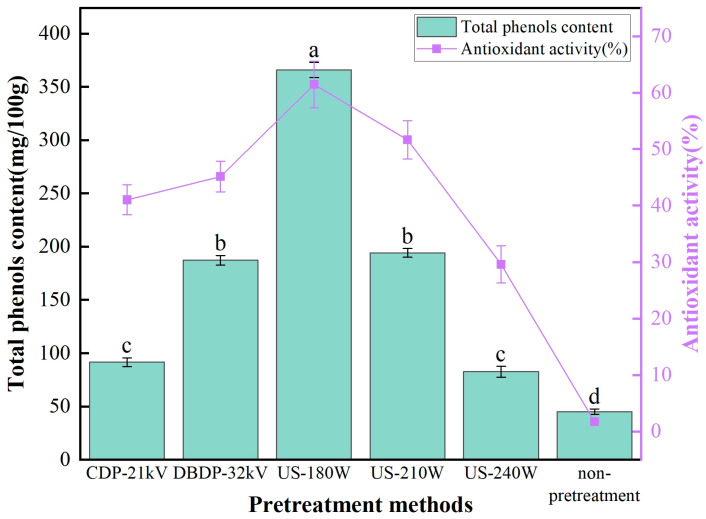
Total polyphenol content and antioxidant efficiency of yam after different non-thermal pretreatments combined with hot-air drying. (Different letters indicate significant differences between sample means).

Total phenol content is an important index to evaluate the quality characteristics of dried materials. Its level can directly reflect the retention of phenolic substances in materials, and phenolic substances have a key impact on the color, flavor, and nutritional value of food. Figure 6 presents the total polyphenol content and antioxidant efficiency of yam after hot-air drying under different pretreatment methods. The data show that the total polyphenol content from high to low is US-180 W > US-210 W > DBDP-32 kV > CDP-21 kV > US-240 W > non-pretreatment. It is worth noting that the total phenol content (TPC) in the 180 W ultrasonic treatment group is the highest, reaching 366 mg/100 g, while with the same pretreatment method (ultrasonic) and power of 240 W, the TPC content drops to 82 mg/100 g, which is relatively low. This difference is directly related to the mechanism of action of the treatments. Ultrasound physically disrupts the cell wall and promotes phenolic release, and its non-thermal nature reduces thermal degradation, with optimal results at a moderate power of 180 W, while high power may exacerbate phenolic oxidation due to overstimulation. CDP treatment oxidizes some phenols due to ionic wind and the high-voltage electric field [18], and DBDP has poor total phenol retention due to phenolic degradation triggered by ozone and free radicals in the plasma [39]. This result proves that ultrasonic pretreatment can preserve phenolic substances well at relatively low power.

As shown in Figure 6, there are significant differences in the effect of different pretreatment methods on the antioxidant efficiency of yam. The purpose of testing antioxidant activity is to evaluate the impact of different pretreatment methods on the retention of phenolic substances in yam and their free radical scavenging ability, so as to determine the optimal drying process. The results of the study showed that the antioxidant activity results were highly consistent with the trend of total phenolic content, and the DPPH radical scavenging rate was significantly higher in the US-180 W group than in the other groups. The core reason why phenolic substances can exert antioxidant effects is that they contain active hydroxyl groups (-OH). Such groups can neutralize reactive oxygen molecules by providing electrons or hydrogen atoms, effectively scavenge free radicals, block oxidation reactions, and thus protect cells from oxidative damage [40]. This mechanism is verified in Newton et al.’s drying experiment on purple cabbage [41]. In the experiment, the total phenol content is significantly positively correlated with the scavenging rate of DPPH and other free radicals, which further confirms the close relationship between phenolic substances and antioxidant capacity.

### 3.6. Low-Field Nuclear Magnetic Resonance (LF-NMR) and Magnetic Resonance Imaging (MRI)

**Figure 7:**
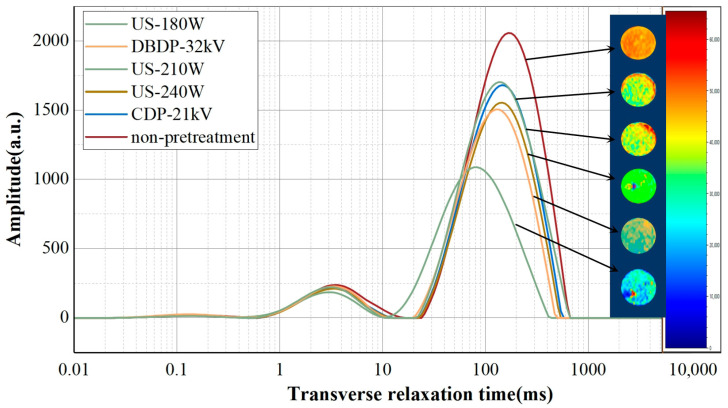
NMR patterns of dried yam after rehydration with different non-thermal pretreatments. (Arrows indicate low-field magnetic resonance imaging images of different groups).

Low-field nuclear magnetic resonance (LF-NMR), as a non-invasive analytical technique, is widely used in the study of moisture status and distribution in food materials. Its core principle is to quantify the mobility of water molecules by measuring the transverse relaxation time *T_2_*, which directly reflects the binding force between hydrogen protons and their freedom of movement [42]. This technique can effectively evaluate the impact of the drying process on moisture migration patterns and the integrity of cellular structures, thus being commonly used in comparative studies of different drying methods. It is worth noting that when yam is over-dried, due to the extremely low moisture content, the hydrogen proton signals become weak, which not only makes the relaxation time (*T_2_*) difficult to accurately detect but also reduces the imaging contrast [43]. Therefore, the test is conducted after rehydration to bring the results closer to the actual state. Figure 7 presents the moisture status and distribution characteristics of yams dried with different pretreatment methods. Under the same test parameters, a higher signal amplitude in the *T_2_* spectrum indicates a higher content of the corresponding type of moisture. Based on the differences in transverse relaxation time, the moisture status in yams can be divided into three categories: *T_21_* (bound water, 0.1–1 ms), *T_22_* (immobilized water, 1–10 ms), and *T_23_* (free water, 10–1000 ms). From the data, it can be observed that with the extension of drying time, the variation amplitudes of *T_22_* and *T_23_* are significantly larger than that of *T_21_*, indicating that the moisture lost during yam drying mainly comes from immobilized water and free water. Meanwhile, the transverse relaxation time shows a left-shift trend, suggesting that the relaxation time is shortened after pretreatment, which means the mobility of moisture in yams is reduced.

During the drying process, the color change in the nuclear magnetic resonance spectrum can directly reflect the concentration of hydrogen protons in the sample—the transition from red to blue corresponds to a decrease in proton concentration from high to low, where a larger red area indicates a stronger corresponding signal intensity [43]. As shown in the MRI images in Figure 7, compared with other pretreatment groups, the yams in the US-180 W treatment group not only have a higher moisture content but also a more uniform moisture distribution. After ultrasonic treatment, the attenuation rate of proton density signals on the cross-section of yams is consistent, indicating that the resistance encountered when moisture diffuses from the central vascular bundle to the peripheral phloem is significantly reduced. This uniform modification of the cellular structure effectively avoids moisture gradient differences caused by tissue heterogeneity in traditional drying, ultimately achieving an overall uniform moisture distribution. During rehydration, the uniform water molecule network allows moisture to permeate into all parts of the yam simultaneously and evenly, reducing cases of insufficient or excessive local rehydration.

### 3.7. Determination of Volatile Organic Compounds

**Figure 8:**
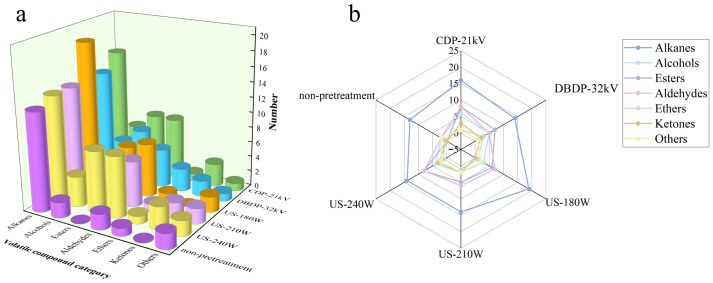
Volatile compounds content in yam: (**a**) content of volatile compounds in yam slices after different non-thermal pretreatments combined with hot-air drying; (**b**) radar plot of volatile compounds content in yam after different non-thermal pretreatments.

Volatile organic compounds are key factors affecting the flavor and quality of dried products, and their types and contents are closely related to drying methods. In this study, headspace solid-phase microextraction–gas chromatography–mass spectrometry (HS-SPME-GC-MS) was used to determine and analyze volatile organic compounds (VOCs) in the samples, focusing on major flavor substances such as aldehydes, alcohols, esters, and hydrocarbons. Orthogonal partial least squares-discriminant analysis (OPLS-DA) was applied to process the data to reveal the influence of different pretreatment methods on volatile components and screen out key differential markers. A total of 55 volatile organic compounds were identified through the NIST database combined with retention time analysis, including 21 alkanes, 7 alcohols, 8 esters, 9 aldehydes, 3 ethers, 3 ketones, and 3 other substances. Figure 8 shows the number and proportion of various volatile components in yams dried with different pretreatments combined with hot-air drying. It can be seen that the number of volatile components in yams dried with different pretreatment methods varies: 43, 38, 40, 32, and 38 volatile components were identified in the CDP-21 kV, DBDP-32 kV, US-180 W, US-210 W, and US-240 W groups, respectively. This phenomenon occurs because hot-air drying accelerates lipid oxidation, the Maillard reaction, etc., due to the high temperature, generating flavor substances such as aldehydes and ketones. Moreover, a long drying time exacerbates the volatilization of heat-sensitive components, and long-term high temperature changes the material structure, affecting the release and retention of volatile components [44].

**Figure 9:**
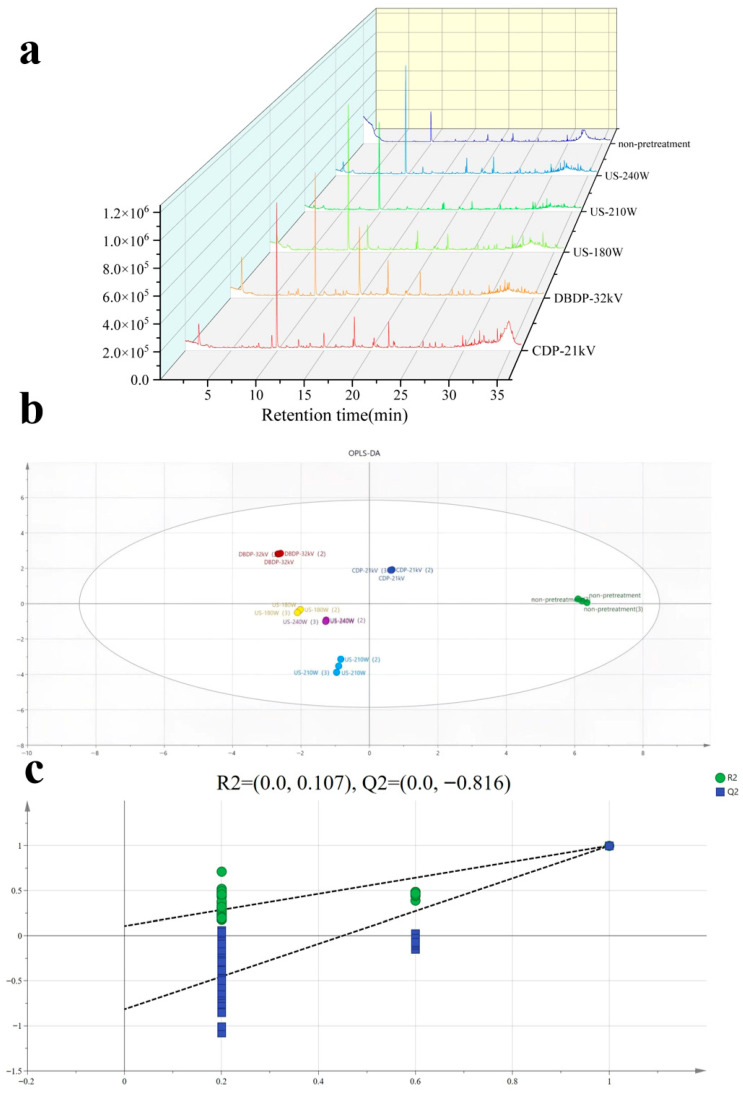
Fingerprint profiles and orthogonal partial least squares discriminant analysis (OPLS-DA) illustrating (**a**) fingerprint profiles of volatile constituents of yam under different pretreatment conditions; (**b**) OPLS-DA analysis of slices of yam with different pretreatments; and (**c**) the results of the model cross-validation.

Figure 9a shows the fingerprint of volatile components in yams dried with different pretreatment methods. The fingerprint is constructed based on retention time and peak area, and the spectral characteristics of different treatment methods are significantly different. Although the types of compounds in each group are similar, there are obvious differences in total content and the ranking of specific components, which, in descending order of content, are aldehydes > alkanes > alcohols > esters > others > ethers > ketones. To explore the differences between samples and screen key components, this study adopted orthogonal partial least squares-discriminant analysis (OPLS-DA), which can establish the relationship between variables and sample components, effectively separate samples from different groups, evaluate the fitting and predictive ability of the model, and be used to analyze differences in volatile components and sample classification [45]. As shown in Figure 9b, in the OPLS-DA model, the independent variable fitting index $R_{x}^{2}$ = 0.996, $R_{y}^{2}$ = 0.999 and the model prediction index $Q^{2}$ = 0.997, where $R_{x}^{2}$ and $R_{y}^{2}$, are used to evaluate the fitting degree and reliability of the model, while $Q^{2}$ is used to evaluate the predictive ability of the model. Values close to 1 indicate that the model has good interpretability and feasibility [46]. To avoid possible overfitting when the OPLS-DA model distinguishes samples, cross-validation can effectively make up for these defects by evaluating generalization ability, identifying overfitting, and optimizing model parameters. After 200 permutation tests, as shown in Figure 9c, the intersection of the Q2 regression line with the vertical axis is less than 0, indicating that the model has no overfitting and is validated, and the results can be used to analyze the differences in yams dried with different pretreatment methods.

The types of volatile compounds detected in yams are relatively few, but their contents are concentrated. A detailed analysis of their odor, content, and form was conducted. Aldehydes are a class of components that contribute significantly to the volatile components of dried yams, and their content and types are closely related to chemical reactions such as lipid oxidation and amino acid metabolism. The nine identified aldehyde compounds in this study are mostly colorless and transparent liquids with fruity and oily aromas, mainly including hexanal, nonanal, decanal, etc. Their low odor thresholds mean that they play a key role in the overall flavor [47]. Different pretreatment methods have a significant impact on aldehyde content. The ultrasonic pretreatment (US) groups, especially the US-180 W group, have higher total aldehyde content than the CDP, DBDP, and non-pretreated groups. The cavitation effect of ultrasound can reduce the damage of high temperature to aldehydes while accelerating moisture migration; in contrast, the high-voltage environment of CDP and DBDP can promote the oxidative decomposition of some aldehydes. Studies have pointed out that high-voltage electric fields can change the redox environment of substances, affecting the stability of aldehydes. The contents of hexanal and nonanal are the highest in the US-180 W group, mainly due to the oxidative degradation of unsaturated fatty acids, while the accumulation of decanal is related to the deamination of amino acids [48].

Alkanes are the most diverse volatile components identified in this study (21 types), with content second only to aldehydes, mainly including dodecane, undecane, and pentadecane. The formation of alkanes is related to the long-chain degradation of lipids and the thermal stability of waxy components, and they usually have low odor activity but play an auxiliary role in the integrity of flavor [49]. The results show that the US-180W group has the highest number and total content of alkanes, among which the relative content of dodecane is significantly higher than that in other groups. Moderate-power ultrasonic pretreatment promotes the release of alkanes from the waxy layer of yam epidermis through mechanical vibration, while avoiding alkane volatilization caused by excessively high power. Relevant studies have shown that when ultrasonic treatment is applied to similar plant samples, appropriate ultrasonic power can effectively destroy cellular structures and promote the dissolution of alkanes in the waxy layer. In contrast, the alkane content in the DBDP-32 kV group is low, so ozone generated during plasma discharge may trigger oxidation reactions of some alkanes [39,50].

Volatile alcohol components are mainly derived from the reduction reaction of aldehydes. Among the seven identified alcohols in this study, 1-octen-3-ol is the most abundant component, with a special herbal aroma and low odor threshold, and is a key contributor to the characteristic flavor of yams [51]. The impact of different pretreatments on alcohols shows a regularity: the total alcohol content in the ultrasonic pretreatment groups is significantly higher than that in the CDP, DBDP, and non-pretreated groups, and shows a trend of first increasing and then decreasing with the increase in power. A moderate ultrasonic cavitation effect promotes the release of alcohols in cells, while excessively high power may cause volatilization or oxidation of alcohols due to local overheating. Studies have found that when ultrasonic treatment is applied to other plant materials rich in alcohols, excessively high ultrasonic power can cause a sharp rise in temperature, leading to the loss of alcohols.

Ester compounds are mostly generated by the esterification reaction of alcohols and acids or derived from the alcoholysis reaction of triglycerides, with significant fruity aromas, and are important components of yam flavor. The eight identified esters in this study include acetic acid, butyl ester, 2-propenoic acid, butyl ester, etc., among which acetic acid and butyl ester have the highest relative content. The results show that the total ester content in the non-pretreated group is higher than that in other pretreated groups, while the ester content in the US, CDP, and DBDP groups is lower. The high-temperature environment of hot-air drying promotes the progress of esterification reactions, while pretreatments such as ultrasound and cold plasma may cause the premature release or hydrolysis of some esters by destroying cellular structures. Relevant studies have shown that high-temperature conditions are conducive to the forward progress of esterification reactions. In addition, the US-180 W group has the most abundant types of esters. It has been found in ultrasonic pretreatment of other plants that low-power ultrasound can promote the release of esters without damaging their structures.

Other types of volatile components identified under different treatment conditions include three ethers, three ketones, and three other substances. Although their total content is relatively low, they still contribute to the complexity and uniqueness of the overall flavor of yams. Ethers usually have special aromatic odors, and their formation is related to the metabolic transformation of phenolic substances or terpenoids in yams [52]. The ethers identified in this study mainly include anisole, etc., and different pretreatment methods have different effects on ether content. The total ether content in the US-180 W group is relatively high because moderate ultrasonic treatment promotes the release of ethers by breaking cells without causing much damage to their structures, while the lower ether content in the DBDP-treated group was attributed to the reaction of reactive particles in the cold plasma with ethers, resulting in their degradation [53]. The ketones in this study include acetone, etc. The results show that the ketone content in the non-pretreated group is slightly higher than that in each pretreated group; this is mainly because pretreatments such as ultrasound and cold plasma change the redox environment inside yams during treatment, inhibiting enzyme-catalyzed reactions related to ketone formation, thereby reducing ketone accumulation [54].

The content changes of the three other types of substances in different pretreatment groups show no obvious regularity, but overall, the types of unclassified substances in the US pretreatment groups are relatively more abundant. At this point, ultrasonic treatment can release various substances from yam cells more comprehensively, while other pretreatment methods have certain limitations in releasing substances with special structures due to differences in treatment intensity or nature [55].

In conclusion, different pretreatments significantly affect the volatile components of hot-air-dried yams. Ultrasonic pretreatment (180 W) through a moderate cavitation effect and mechanical vibration effectively releases intracellular contents and reduces structural damage, with the best effect on retaining and promoting key flavor substances such as aldehydes, alkanes, alcohols, and esters. In contrast, cold plasma treatments such as corona discharge plasma (CDP) and dielectric barrier discharge plasma (DBDP), due to causing changes in the redox environment or generating active particles, mostly have degradation or inhibition effects on volatile components. Studies have shown that the rational selection of pretreatment methods is the key to achieving directional regulation of yam flavor quality.

### 3.8. Correlation

**Figure 10:**
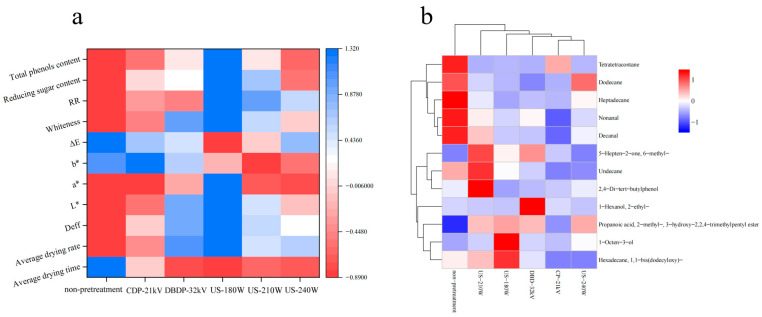
Statistical analysis. (**a**) Heat map of correlation between different drying methods and drying indexes; (**b**) heat map of clustering for screening of volatile substances in yam under different pretreatment conditions.

Correlation analysis was conducted after normalizing the drying characteristic data (average drying rate, average drying time, rehydration rate, and color difference value) of yam under different drying conditions. Figure 10a shows a correlation heatmap of the impact of different drying methods on the drying indexes of yam. The analysis results show that drying rate is significantly positively correlated with effective moisture diffusion coefficient; this correlation suggests that the degree of disruption of the cellular structure by the pretreatment directly affects the water migration capacity, which in turn determines the drying efficiency, which explains the optimal drying rate exhibited by the US-180 W group due to the higher effective water diffusion coefficient, indicating that enhancing moisture migration capacity through pretreatment can directly improve drying efficiency. The rehydration rate is positively correlated with drying rate, L* value, and whiteness and negatively correlated with total color difference *ΔE*, suggesting that the intact retention of cellular structures is beneficial to moisture reabsorption and browning inhibition. Total phenol content is extremely positively correlated with antioxidant activity, reducing sugar is positively correlated with total phenol, and the US-180 W group performs best in reducing sugar, total phenol, antioxidant activity, drying rate, and rehydration rate, reflecting the synergistic advantage of efficient drying and nutrient retention. Therefore, the US-180 W treatment group was statistically significant compared to the other methods. Figure 10b demonstrates the clustering heat map for screening differences in volatile components of yam under different pretreatment conditions. The effects of different pretreatment methods on the flavor substances were presented visually. From the clustering results, the US-180 W group showed a significant difference in the volatile components profile with higher content of aldehydes and alkanes than the other groups, while the CDP-21 kV and DBDP-32 kV groups and the unpretreated group showed lower content and closer clustering of the characteristic flavor substances. This clustering pattern is consistent with the correlation analysis of the drying indexes, further confirming the advantage of US-180 W pretreatment in retaining flavor substances, which is synergistic with the group’s excellent performance in color, rehydration effect, and nutrient content, supporting its comprehensive value in drying efficiency and quality optimization.

## 4. Conclusions

This study compared the effects of non-thermal pretreatments such as corona discharge plasma (CDP-21 kV), dielectric barrier discharge plasma (DBDP-32 kV), and ultrasound with different powers (US-180 W, 210 W, 240 W) combined with hot air on the drying characteristics and quality of yams. The results showed that all pretreatments could improve drying efficiency. Among them, the US-180 W pretreatment had the highest average drying rate, which was 70.6% higher than that of the non-pretreated group, the largest effective moisture diffusion coefficient, and the optimal rehydration rate. This is related to the microchannels formed by the ultrasonic cavitation effect, which promote moisture migration. In terms of quality, the US-180 W group had the highest brightness (L*) and redness (a*) and the smallest color difference (ΔE), which could better retain the original color; in terms of nutritional quality, its total phenol content and antioxidant activity were significantly higher than those of other groups, and the reducing sugar content was 36.58 and 31.17% higher than that of the CDP-21 kV and DBDP-32 kV groups, respectively. Low-field nuclear magnetic resonance showed that the moisture distribution after rehydration was more uniform. In total, 55 compounds were identified in the analysis of volatile components, and the US-180 W group had more abundant types and contents, mainly alkanes and aldehydes. Of particular importance, energy consumption analysis showed that while achieving efficient drying and high-quality retention, the total energy consumption of the US-180 W group was significantly lower than that of the cold plasma pretreatment groups and other ultrasonic groups. Its energy-saving advantage mainly comes from the fact that ultrasonic pretreatment, under moderate power, can not only efficiently destroy cellular structures through cavitation effect to accelerate moisture migration and reduce hot-air drying time but also avoid the sharp increase in energy consumption during the pretreatment stage caused by excessively high power, at the same time saving more energy than the high-voltage discharge process of cold plasma. In conclusion, ultrasonic 180 W pretreatment is comprehensively optimal in terms of drying efficiency, quality retention, and energy conservation, providing an experimental reference with high efficiency, high quality, and energy conservation for optimizing the drying process of foods such as yams.

## Data Availability

The original contributions presented in the study are included in the article, further inquiries can be directed to the corresponding author.

## Abbreviations

The following abbreviations are used in this manuscript:

CDP	Corona discharge plasma
DBDP	Dielectric barrier discharge plasma
US	Ultrasonic waves
HAD	Hot-air drying
RR	Rehydration rate
LF-NMR	Low-Field Nuclear Magnetic Resonance
GC-MS	Gas chromatography–mass spectrometry
OPLS-DA	Orthogonal partial least squares discriminant analysis
TPC	Total phenol content
